# *Ficus hirta* Vahl. Ameliorates Nonalcoholic Fatty Liver Disease through Regulating Lipid Metabolism and Gut Microbiota

**DOI:** 10.1155/2022/3474723

**Published:** 2022-05-10

**Authors:** Ting Quan, Fangyu Zhou, Huiyuan Chen, Lina Jian, Yuxuan Yang, Fan Xia, Shijian Xiang, Benjie Zhou, Shasha Li

**Affiliations:** ^1^Department of Pharmacy, The First Affiliated Hospital of Jinan University, Guangzhou 510632, China; ^2^School of Pharmacy, Guangdong Medical University, Dongguan 523808, China; ^3^Department of Pharmacy, The Seventh Affiliated Hospital, Sun Yat-sen University, Shenzhen 518107, China

## Abstract

Nonalcoholic fatty liver disease (NAFLD) has gradually become one of the most serious liver diseases threatening human health in the world. Currently, Chinese herbal medicine is a potentially important treatment option for NAFLD, and the development of effective Chinese herbal medicine has a good prospect. Previous studies have suggested that *Ficus hirta* Vahl. (FV) has various protective effects on the liver. In this study, we investigated the therapeutic outcomes of FV treatment for the liver disease and its underlying mechanism using HepG2 cell lines induced by palmitate (PA) and mouse model fed with high-fat diet (HFD). FV mainly exerts pharmacological effects by mediating lipid metabolism and inflammation. During the lipid metabolism regulation process, CD36, SREBP-1, SCD1, PPAR *γ*, ACOX1, and CPT1*α* are the key factors related to the healing effects of FV on NAFLD. During the inflammation process, the downregulation of IL-6, IL-1*β*, and TNF-*α* is involved in alleviation of NAFLD. Furthermore, CD36 overexpression promotes lipid abnormal metabolism and inflammation in PA-induced HepG2 cells, while CD36 knockdown and FV supplementation reverse these responses. In addition, FV also modulates gut microbiota composition, such as *Allobaculum*, *Faecalibaculum*, and *Butyricicoccus* in HFD-fed mice. In summary, our findings demonstrated that FV exerted a beneficial preventive and therapeutic effect on NAFLD by improving lipid metabolism and inflammation as well as regulating the structure of gut microbiota, and therefore, FV may be a candidate for the treatment of NAFLD.

## 1. Introduction

Nonalcoholic fatty liver disease (NAFLD) includes a wide range of liver damage, including NAFL (simple steatosis; NAFL), nonalcoholic steatohepatitis (NASH) with inflammation and hepatocyte injury, and advanced fibrosis and cirrhosis [1]. The prevalence of NAFLD in the general population is estimated to be 25% worldwide [2]. The development of NAFLD is closely related to the abnormal lipid metabolism; variations in intracellular cholesterol transport and imbalance of cholesterol homeostasis in NAFLD can cause the accumulation of hepatic free cholesterol [3]. The imbalance between lipid intake and disposal leads to the accumulation of hepatic fat [4]; in addition, NAFLD is often accompanied by a high risk of type 2 diabetes and cardiovascular disease [5]. Furthermore, several studies have demonstrated the close association between gut microbiota and the occurrence and the development of NAFLD. There may be a possible direct association between gut microbiota and inflammation [6]. Therefore, it is generally believed that NAFLD is a liver manifestation of metabolic syndrome (MS) [7]. Nonalcoholic steatohepatitis (NASH) is a subtype of NAFLD and has the potential to progress and result in liver fibrosis, cirrhosis, hepatocellular carcinoma (HCC), and liver transplantation [8]. NASH-related complications bring severe physical, economic, and patient experience pressures to patients, families, and society [9]. Unfortunately, there are limited approaches available for the treatment of NAFLD other than lifestyle changes and reduction of high-calorie, low-fiber diets. Therefore, it is urgent to develop pharmacologically approved treatments for NAFLD [10].

CD36 (cluster of differentiation 36) is a scavenger receptor, which acts as a promoter of transport and uptake of the oxidized low-density lipoprotein (ox-LDL) and long-chain fatty acids [11]. The uptake of fatty acid by the liver may be promoted by fatty acid transport proteins (FATPs) and FAT/CD36 (fatty acid translocase), which have been reported to be higher in obese individuals and NAFLD patients [12]. Oxidized LDL and fatty acid bind to alpha helix at the exposed distal end of the CD36 membrane. CD36 is a lipid-binding pocket containing key Lys164 that initiates downstream signal transduction or promotes fatty acid binding and internalization [13]. Currently, the studies have shown that CD36 is an important factor in liver injury associated with metabolic diseases. In the condition of high expression of CD36, a large amount of fatty acid can be synthesized and then promote the development of fatty liver [14]. The stimulation by palmitate can lead to the elevation of the expression of CD36 in HepG2 cells and the formation of a lipid droplet, which facilitates fatty acid uptake and lipid accumulation [15]. When the liver is exposed to excessive fatty acid for a long period, the function of CD36 will be dysregulated, which may reflect the increased palmitoylation of hepatic CD36. This phenomenon has been reported to be related to NAFLD [16].

*Ficus hirta* Vahl. is a traditional food and medicinal material in southern China. The water extracts of its roots have been widely used clinically in the treatment of NAFLD [17]. Previous studies have focused on the chemical investigation of the roots of *Ficus hirta* Vahl. The predominant chemical constituents of *Ficus hirta* Vahl. mainly include psoralen, bergapten, luteolin, and apigenin [18]. Several studies have shown the pharmacological activities of *Ficus hirta* Vahl., such as antioxidation [19], anti-inflammation, analgesic, antitussive, antiasthmatic [20], and hepatoprotective [21]. In addition, *Ficus hirta* Vahl. is effective to prevent the occurrence of alcohol-induced hepatic damage in mice via scavenging free radical inhibiting lipid peroxidation [22]. These results have revealed that extracts of *Ficus hirta* Vahl. can be used as a candidate drug for liver protection for the treatment of liver diseases.

To clarify the role of *Ficus hirta* Vahl. (FV) in the treatment of NAFLD, we studied the effects of FV on lipid metabolism and inflammation in NAFLD using the HepG2 cell line induced by PA and the mouse model fed with HFD and determined the therapeutic potential of FV. More importantly, we also found that *Ficus hirta* Vahl. can alleviate abnormal lipid anabolism and improve inflammation via downregulating the expression of CD36. Meanwhile, *Ficus hirta* Vahl. relieved liver inflammation in HFD-fed mice by changing gut microbiota component.

## 2. Materials and Methods

### 2.1. Materials and Reagents

*Ficus hirta* Vahl. was bought from Kangmei Pharmaceutical Co., Ltd. (Guangdong, China); palmitic acid (PA, P9697) was bought from Sigma-Aldrich (St. Louis, MO, USA); primary antibodies against IL-6 (DF6078, 1 : 1500), IL-1*β* (AF5103, 1 : 1500), TNF-*α* (AF7014, 1 : 500), SREBP-1 (AF6283, 1 : 1500), CPT1*α* (DF12004, 1 : 1500), ACOX1 (DF12046, 1 : 1500), and GAPDH (AF7012, 1 : 2000) were purchased from Affinity Biosciences Co., Ltd. (Jiangsu, China); antibody against CD36 (18836-1-AP, 1 : 1000) was bought from Proteintech (Wuhan, China); HMGCR (ab171830, 1 : 5000) was purchased from Abcam (Shanghai, China); RIPA buffer and BCA protein assay kit were bought from Beyotime Biotechnology (Shanghai, China); the high-fat diets (HF60), including 60% fat, 20% carbohydrate, and 20% protein, were purchased from Dyets (Dyets Biotechnology Co., Ltd., USA); the normal fat diets including 20 kcal % fat were purchased from Guangdong Medical Laboratory (Guangzhou, China); and D-(+)-glucose (CAS: 50-99-7) and fructose (CAS: 7660-25-5) were bought from Macklin Biochemical Co., Ltd. (Shanghai, China).

### 2.2. Preparation and Identification of FV

The root of *Ficus hirta* Vahl. (FV) was extracted by purified water. The components of FV were detected by UPLC-Q/TOF-MS/MS (X500R, AB SCIEX, USA). The sample was separated by an UPLC C18 analytical column with the size of 2.1 mm × 100 mm, I.D. 1.8 *μ*m (ACQUITY UPLC®HSS T3, Waters, USA). In the modified method, the binary gradient mobile phase with water (0.05% acetic acid) was used as mobile phase A, and acetonitrile (ACN) was used as mobile phase B. Supplementary Table 1 shows the time program of the gradient elution. The sample injection volume was 5 *μ*L. Mass spectrometric analysis was conducted by a SCIEX X500R, which was equipped with electrospray ionization (ESI) Turbo V™ ion source, operating in positive and negative ion modes. Supplementary Table 2 lists the parameters in the method. We performed qualitative and relative quantitative analysis using SCIEX O.S. software V 2.0 (AB SCIEX).

### 2.3. Cell Culture

HepG2 cells (human liver carcinoma cell line) were obtained from Cell Resource Center, Shanghai Institute for Biological Sciences, Chinese Academy of Sciences (Shanghai, China). The obtained HepG2 cells were incubated in Dulbecco's modified Eagle's medium (DMEM; Gibco, Grand Island, NY) supplemented with 10% fetal bovine serum (FBS, Gibco, United States), in which the concentration of penicillin was 100 U/mL and the concentration of streptomycin was 100 U/mL, under the condition of 37°C and 5% CO_2_. HepG2 cells were incubated with 0.25 mM PA to establish cell model of lipid accumulation and were supplied with different doses of FV extract (high-dose 30 mg/mL, low-dose 15 mg/mL, raw medicinal material content) for 24 h.

### 2.4. Animals and Experimental Design

C57BL/6J mice (male, eight-week, weight of 18-25 g) were obtained from the Experimental Animal Centre, Guangdong Medical Laboratory (Guangzhou, China). All subjects were acclimated in a 12 h light/dark cycle in pathogen-free (SPF) laboratory in a controlled environment (temperature: 25 ± 2°C, humidity: 50-75%) and fed with a certified laboratory diet *ad libitum*. The mice were provided with tap water *ad libitum*. After 2 weeks of habilitation, we randomly divided the mice into 4 groups: the NFD group (*n* = 8) was fed with a normal fat diet; the HFD group (*n* = 10) and FV treatment groups (FV-L, FV-H group, *n* = 10 each group) were fed with high-fat diet and aqueous solution of glucose and fructose for 17 weeks to establish a nonalcoholic fatty liver disease model. The animals in the FV-L and FV-H groups were administered with the water extract of FV at the doses of 5 g/kg and 10 g/kg (raw medicinal material content), respectively. The other groups were administrated with equivalent amount of saline. Body weight was measured once per week. At the end of the experiment, mice were fasted for 18 hours and anesthetized with a 1% pentobarbital (50 mg/kg BW) via intraperitoneal injection. We collected blood samples from the subjects eyeballs and centrifuged the sample at 3000 rpm and 4°C for 15 min to collect serums. In addition, liver tissues of all animals were collected and stored at -80°C for later use. All the animal studies were in accordance with the relevant national legislation and local guidelines on the ethical use of animals. In addition, all the procedures in the study have been approved by the Institutional Animal Care and Use Committee of Jinan University.

### 2.5. Hematoxylin-Eosin (H&E) and Oil Red O Staining

Oil red O was used to stain the HepG2 cells according to the instruction of Solarbio kits (Cat# G1262, Beijing, China). Three images per sample were taken using an optical microscope (Nikon, Shanghai, China). Then, the tissue sections were performed for H&E staining and oil red O staining according to the instruction of Solarbio kits (Cat# G1262, Beijing, China).

### 2.6. Biochemical Analysis

The concentration of total cholesterol (TC, A111-1-1), triglyceride (TG, A110-1-1), alanine aminotransferase (ALT, C009-2-1), aspartate aminotransferase (AST, C010-2-1), and low-density lipoprotein cholesterol (LDL-C, A113-1-1) of the mouse serum samples was determined according to the instruction of diagnostic kits from Nanjing Jiancheng Bioengineering Institute (Nanjing, China).

### 2.7. Western Blot Analysis

Total protein was extracted from liver tissue and hepatocytes by RIPA lysis, and its concentration was measured by a BCA protein assay kit. Western blot analysis was routinely conducted with primary antibodies against GAPDH, IL-6, IL-1*β*, TNF-*α*, SREBP-1*α*, HMGCR, ACOX1, CD36, and CPT1*α*. 30 *μ*g protein was placed in each well. An ECL (Affinity Biosciences LTD, Jiangsu, China) was used to detect the protein bands. The band intensity by densitometry for quantification was evaluated using ImageJ 1.48 analysis software and expressed as the mean area density.

### 2.8. Determination of Inflammatory Cytokine Levels in Serum

The levels of IL-6 (EMC004), IL-1*β* (EMC001b), and TNF-*α* (EMC102a) in each mouse serum sample were determined by ELISA kits, which were produced by Neobioscience (Shenzhen, China).

### 2.9. RT-qPCR Analysis

The gene expression of HepG2 cells and mouse liver tissues was performed by RT-qPCR. Trizol regent was used to isolate the total RNA of the HepG2 cells and liver tissue, and HiScript II Q RT SuperMix for qPCR (cat no. R223-01, Vazyme, Nanjing, China) was used to reverse transcribe them into cDNA. The synthesized cDNA was used as a template and quantified using the BioEasy Master Mix Kit (cat no. BSB25L1B, SYBR Green, High ROX) and a real-time PCR detection system (Line Gene 9600 Plus, Bioer Technology, China). Supplementary Table 3 lists the human and mouse primer sequences for quantitative real-time PCR.

### 2.10. RNA-seq, KEGG Analysis, and Gene Set Enrichment Analysis

The transcriptome sequencing was performed by Novogene (Beijing, China). After generating clusters, we sequenced the library preparations on the Illumina Novaseq 6000 platform.

In order to analyze Kyoto Encyclopedia of Genes and Genomes (KEGG) as well as Gene Ontology (GO) biological process, the crossover differentially expressed genes (DEGs, *P* < 0.05 and |log_2_FC| > 2.0) were submitted into the Web-based gene set analysis toolkit (WebGestalt, http://www.webgestalt.org/option.php) [23]. Then, the Gene Set Enrichment Analysis (GSEA) was conducted on the Java GSEA platform. We calculated the fold change of gene expression, and the gene list was generated according to the change of |log_2_FC|. The genes involved in each KEGG pathway were denoted as a gene set. Then, a ranked list and a “gene set” permutation type of the gene set was generated. *P* < 0.05 was set as the cutoff criterion.

### 2.11. Cell Transfection

The plasmid overexpressing CD36 was designed and built by iGene Biotechnology Co., Ltd. (Guangzhou, China) to generate a CD36-overexpressed (CD36 OE) cell line, and an empty vector was used as a control. siCD36 was synthesized by RIBOBIO Co., Ltd. (Guangzhou, China). The experiment of cell transfection was performed. The efficiency of knockdown and overexpression of CD36 were confirmed by western blot analysis and RT-qPCR.

### 2.12. 16S rRNA Sequencing

Frozen mouse fecal samples were used to characterize the gut microbiota. The genomic DNA was extracted and used to amplify the V3–V4 region of the 16S rRNA genes. First, the amplicons were purified and then combined in equal amounts for sequencing library preparation and Miseq sequencing analysis. The PE reads were obtained from Miseq sequencing, and then, they were spliced based on the overlapping relationship. Next, the sequence quality was simultaneously controlled and filtered. The samples were distinguished and then went through OTU cluster analysis and species taxonomy analysis. Based on the taxonomic information, the community structure can be statistically analyzed at different taxonomic levels. Multiple samples were used in various statistical and visual analyses to study the community composition and phylogenetic information, such as multivariate analysis and significance of differences tests. In addition, we performed Kyoto Encyclopedia of Genes and Genomes (KEGG) enrichment analysis to further study the biological signaling pathways in the gut microbiota.

### 2.13. Spearman's Analysis

The correlation between the 8 genera of microbiota and 4 signaling pathways was analyzed using Spearman's correlation coefficient (*r*). In the correlation analysis, *P* < 0.05 was considered as the significant criterion.

### 2.14. Statistical Analysis

The data was expressed as the mean ± standard deviations (SD). Furthermore, statistical analysis was implemented using GraphPad Prism 5.0 software (GraphPad Software, Inc.; San Diego, CA, USA). The statistical analyses in this study including one-way ANOVA and Tukey's post hoc test were used to compare multiple groups. In the statistical test, *P* < 0.05 was considered statistically significant.

## 3. Results

### 3.1. Identification of Bioactive Components for FV Extract

**Figure 1:**
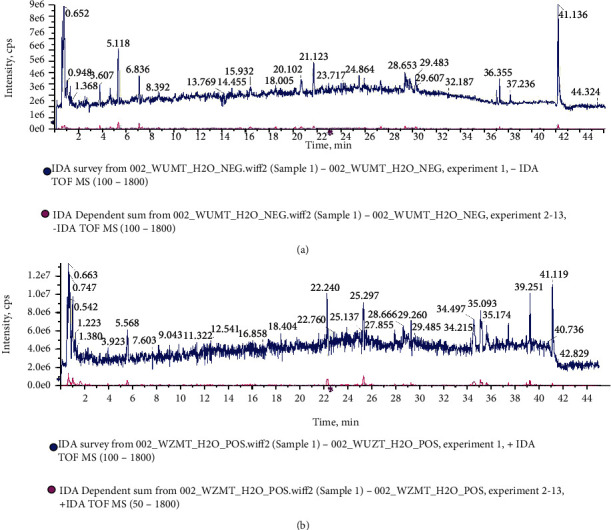
UPLC-Q/TOF-MS/MS analysis. The total ion chromatogram (TIC) of FV of by UPLC-Q/TOF-MS/MS in the (a) negative and (b) positive ion modes.

The total ion chromatogram (TIC) of FV was investigated by UPLC-Q/TOF-MS/MS. The results are shown in Figure 1. The components were characterized by matching with SCIEX high-resolution MS/MS database or ChemSpider online database. The results identified a total of 54 chemical constituents in FV. Among them, 23 ingredients were determined in positive ion mode, while 31 ingredients were determined in negative ion mode. The FV extract contained 11 types of coumarins, 11 types of flavonoids, 5 types of carboxylic acids, 3 types of terpenes, 6 types of aldehydes, and other types of compounds. The detailed information is shown in Table 1.

### 3.2. Amelioration of Lipid Accumulation and Inflammation by FV In Vitro

**Figure 2:**
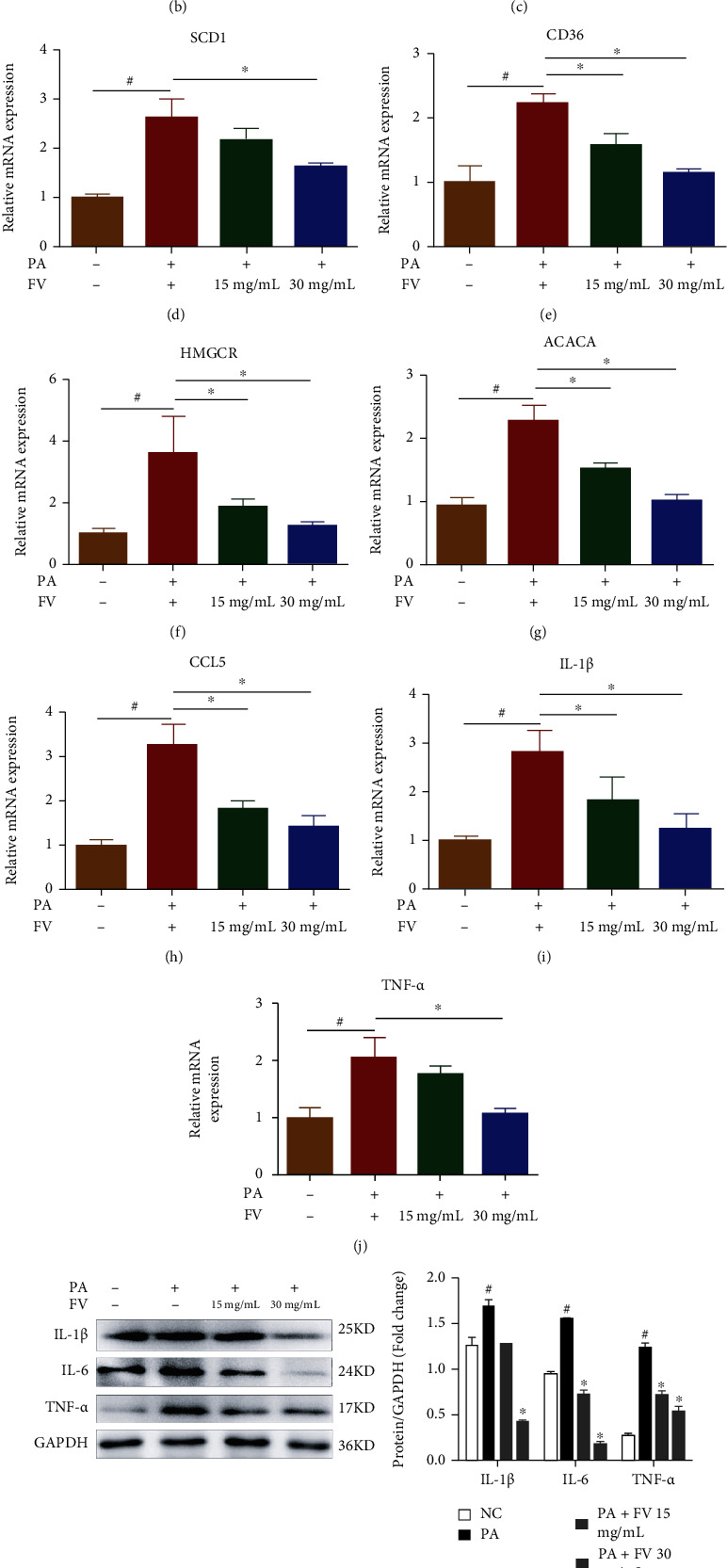
Amelioration of lipid accumulation in PA-induced HepG2 cells by FV. (a) Oil red O was used to measure the level of lipid accumulation (magnification 100x, scale bar = 250 *μ*m). (b) The oil red O-positive area was analyzed and quantified. (c–j) The relative mRNA expression levels of *FABP1*, *SCD1*, *CD36*, *HMGCR*, *ACACA*, *CCL5*, *IL-1β*, and *TNF-α* were determined by qRT-PCR. (k) The proinflammatory factor protein levels of IL-1*β*, IL-6, and TNF-*α*. (l) The protein levels related to lipid metabolism of SREBP-1, ACOX1, CD36, CPT1*α*, and HMGCR were analyzed by western blotting, and the relative ratios were calculated and expressed as the mean ± SD; *n* = 3. ^#^*P* < 0.05 means that the difference between the NC group and the PA group is significant. ^∗^*P* < 0.05 means that the difference between the PA group and the FV (30 mg/mL) group or the FV (15 mg/mL) group is significant.

We used oil red O staining to explore the effect of FV on lipid homeostasis in PA-induced HepG2 cells. As shown in Figure 2(a), PA caused a significant increase in the number of lipid droplets in HepG2 cells, while FV reversed it in a dose-dependent manner; the oil red O score is showed in Figure 2(b). Furthermore, western blot and RT-qPCR were performed to determine the protein and mRNA expression levels of biomarkers related to lipogenesis and inflammation in HepG2 cells. The results demonstrated that after FV treatment, the expression levels of HMGCR (Figures 2(f) and 2(l)), SREBP-1 (Figure 2(l)), FABP1 (Figure 2(c)), SCD1 (Figure 2(d)), CD36 (Figures 2(e) and 2(l)), and ACACA (Figure 2(g)) were remarkably suppressed while the levels of key enzymes regulating fatty acid oxidation (ACOX1 and CPT1*α*) (Figure 2(l)) were increased. Meanwhile, we found that FV treatment significantly ameliorated inflammation in the HepG2 cell line induced by PA by decreasing the level of proinflammatory factor, including IL-1*β* (Figures 2(i) and 2(k)), IL-6 (Figure 2(k)), TNF-*α* (Figure 2(k)), and CCL5 (Figure 2(h)). These results suggested that FV might restore PA-induced lipogenesis and inflammation responses.

### 3.3. Attenuation of Hepatic Steatosis and Serum Lipid Levels in HFD-Fed Mice by FV

**Figure 3:**
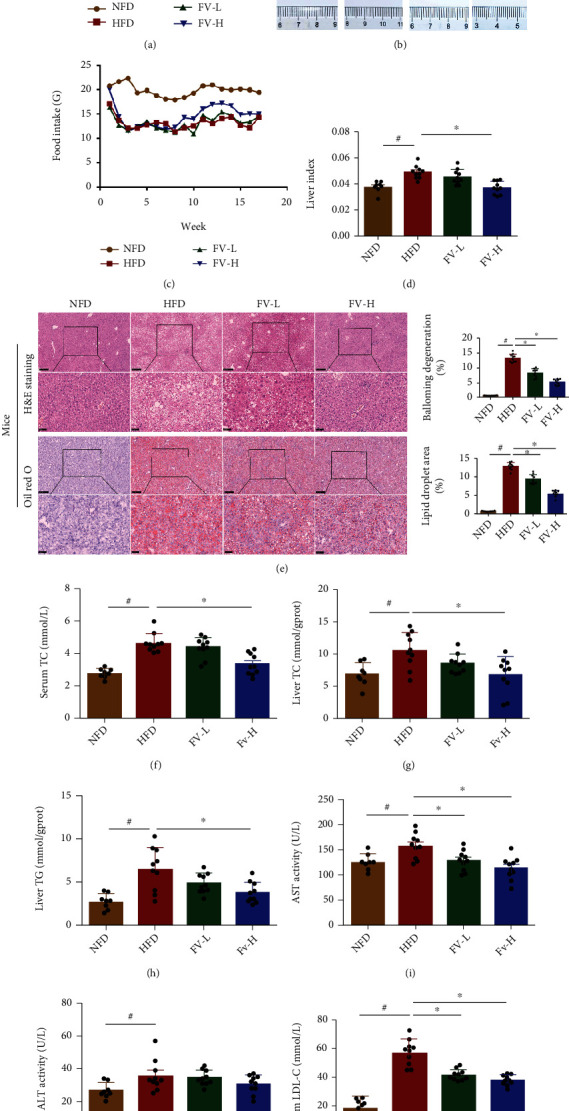
Effects of FV on accumulation of liver fat and lipid levels in serum in HFD-fed mice. (a) Body weight, (b) macroscopic observation of livers in subjects in different groups, (c) weekly food intake per mouse in each group, (d) liver index, (e) light microscopic H&E image and oil red O staining image of liver tissues of subjects in different groups (x100 original magnification, scale bar = 250 *μ*m; 400x original magnification, scale bar = 50 *μ*m), H&E score and oil red O score, (f) serum TC, (g) liver TC, (h) liver TG, (i) serum AST, (j) ALT, and (k) serum LDL-C. The data are expressed in the format of mean ± SD (*n* = 8, 10, 10). ^#^*P* < 0.05 means there is a significant difference between the NFD group and the HFD group. ^∗^*P* < 0.05 means the difference between the HFD group and the FV-L group or the FV-H group is significant. NFD: normal fat diet group; HFD: high-fat diet group; FV-L group: high-fat diet with FV administration at dose of 5 g medicinal materials/kg body weight; FV-H group: high-fat diet with FV at dose of 10 g medicinal materials/kg body weight.

A mouse model fed with HFD was utilized to further explore the beneficial effects of FV. As shown in Figures 3(a) and 3(c), HFD-fed mice exhibited an increased body weight and a higher liver index, and a 17-week FV administration alleviated these gains. Compared with normal diet mice, HFD-fed mice showed liver enlargement and discoloration, which were much improved by the FV administration (Figure 3(b)).

To evaluate the extent of liver injury in mice, we performed H&E staining and measured transaminase level in mouse serum. The liver tissue of mice in the HFD group showed obvious changes in morphology, including extensive cell necrosis, loss of hepatic structure, and a large amount of inflammatory cell infiltration. However, compared with the HFD group, the prophylactic use of FV obviously rescued the injured area in a dose-dependent manner (Figure 3(d)). In addition, compared to the mice fed with high-fat diet, the serum ALT and AST levels in the FV treatment group were decreased (Figures 3(i) and 3(h)).

As shown in oil red O staining, the accumulations of lipid droplets in hepatocytes were significantly severe in the HFD group, which were reduced in the liver of mice with FV administration (Figure 3(d)). Furthermore, compared with the HFD group, the increased levels of TC and TG in the liver of mice with FV treatment were much recovered (Figures 3(f) and 3(g)); similarly, the increased level of TC and LDL-C in serum was also removed by FV treatment (Figures 3(e) and 3(j)).

### 3.4. Attenuates Lipogenesis and Inflammation in the Liver of HFD-Fed Mice by FV

**Figure 4:**
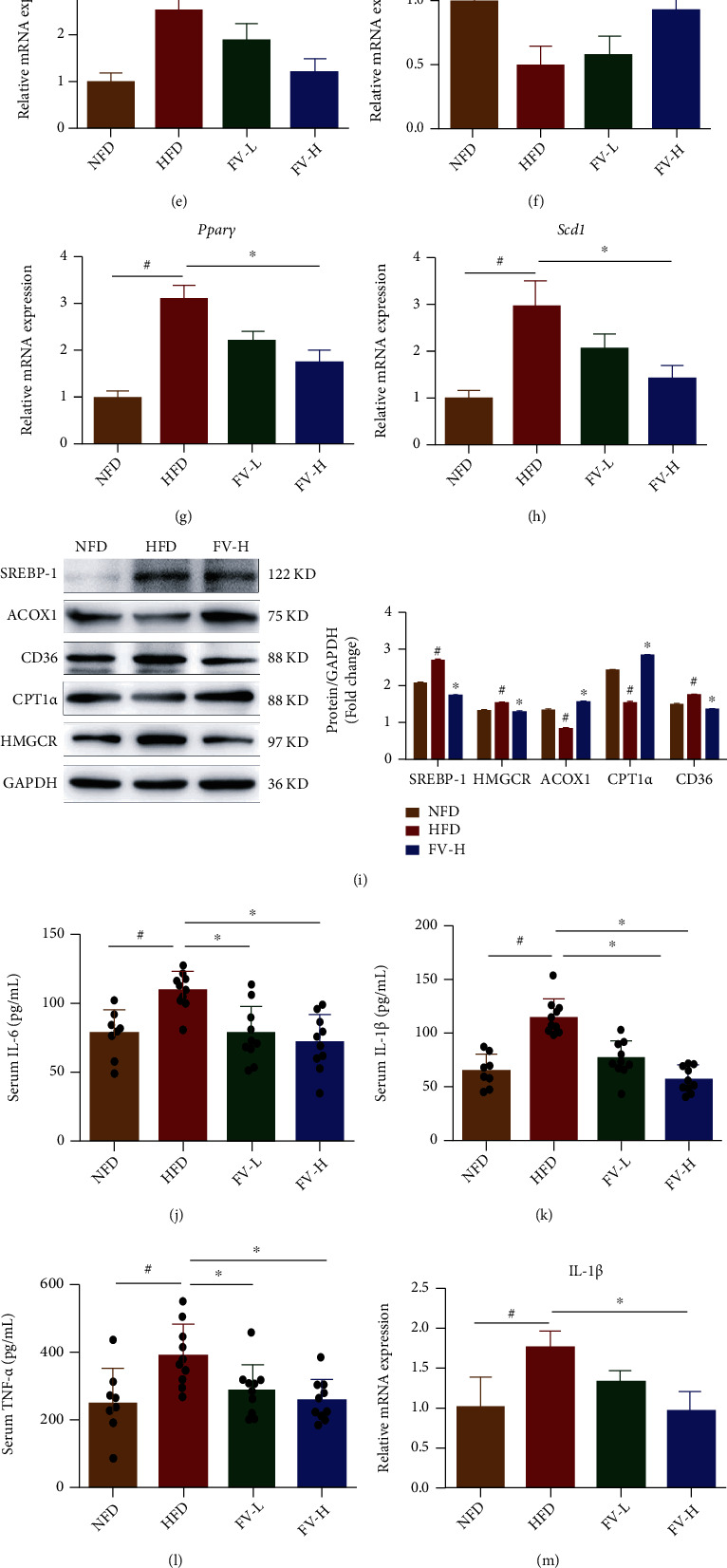
Effects of FV on liver lipogenesis-related markers in the mice fed with HFD. (a–h) The relative mRNA expression levels of *Acaca*, *Cd*36, *Cpt*1*α*, *Srebp-*1, *Hmgcr*, *Pparα*, *Pparγ*, and *Scd*1 were determined by qRT-PCR. (i) The lipid metabolism relevant protein levels of SREBP-1, ACOX1, CD36, CPT1*α*, and HMGCR were analyzed by western blotting, and the relative ratios were calculated. (j–l) Serum IL-6, IL-1*β*, and TNF-*α* were identified by ELISA kits. (m, n) The relative mRNA expression levels of IL-1*β*, TNF-*α*, and Ccl5 were identified by qRT-PCR. (p) The proinflammatory factor protein levels of IL-1*β*, IL-6, and TNF-*α* were analyzed by western blotting, and their relative ratios were calculated. The densitometry was obtained by averaging repeated experiments results, normalized to GAPDH. The data are expressed as the mean ± SD, *n* = 3. ^#^*P* < 0.05 means there is significant difference between the NFD group and the HFD group. ^∗^*P* < 0.05 means the difference between the HFD group and the FV-L group or the FV-H group is significant.

To analyze the influencing mechanisms of FV on the accumulation of lipid, we further studied the beneficial effects of FV in the *in vivo* model. Compared with HFD-fed mice, FV treatment significantly reduced the mRNA expression of Srebp-1, Acaca, Hmgcr, Fabp1, Ppar*γ*, and Cd36. Meanwhile, FV treatment led to the increase (Figures 4(a)–4(h)) in the level of key enzymes related to the regulation of fatty acid oxidation (Ppar*α* and Cpt1*α*) (Figures 4(c) and 4(f)). It is worth noting that compared with the mice fed with HFD, FV treatment caused the reduction of the expressions of lipid metabolism-related proteins, which included SREBP-1, HMGCR, and CD36. However, ACOX1 and CPT1*α* levels in the livers of animals with NAFLD were increased after FV treatment (Figure 4(i)).

ELISA results showed that compared with HFD-fed mice, FV significantly downregulated the levels of IL-6, IL-1*β*, and TNF-*α* in serum (Figures 4(j)–4(l)). As shown in Figure 4(p), the upregulated proteins of TNF-*α*, IL-6, and IL-1*β* in the liver tissues in the mice fed with HFD were also suppressed by FV administration. In addition, the mRNA expression of Tnf-*α*, Il-1*β*, and Ccl5 in liver tissues was successively suppressed by FV administration (Figures 4(m)–4(o)). These data indicated that FV partially alleviated the hepatocyte damage by reducing inflammation in serum and hepatocytes.

### 3.5. FV Regulated Gene Expression and Signaling Pathways in the Liver of HFD-Fed Mice

In order to systematically investigate the potential mechanism of FV on NAFLD mice, we conducted transcriptome analysis through RNA sequencing of liver tissues in the mice fed with NFD and the mice fed with HFD with or without FV treatment.

**Figure 5:**
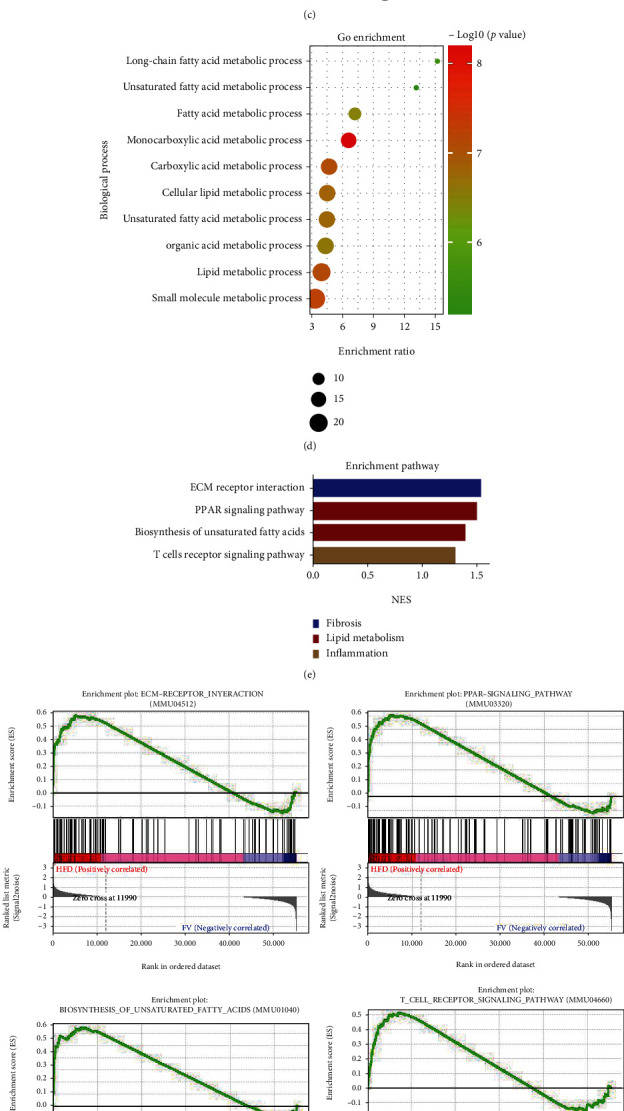
Key targets and pathway between the NFD group, the HFD group, and the FV-H group revealed by RNA-seq analyses. (a) PCA of the RNA-seq data from the NFD group, the HFD group, and the FV-H group. (b) Volcano plot indicating the DEGs (red, upregulated genes; green, downregulated genes) from three groups (HFD group vs. NFD group or FV-H group). (c) The KEGG pathway enrichment analysis of crossover DEGs. (d) The GO enrichment analysis of crossover DEGs. (e, f) GSEA pathway enrichment analysis of pathways that are related to inflammation, lipid metabolism, and fibrosis. (g) Heat map of gene expression profiles related to lipid metabolism, fibrosis, and inflammation based on RNA-seq dataset. *n* = 3 in each group.

The results from principal component analysis (PCA) indicated that the gene expression profile of mice from the FV group clustered with the NFD group but separated from the HFD group (Figure 5(a)). The volcano plots demonstrated that the differentially expressed genes (DEGs) in the HFD group underwent significant changes compared to those in the NFD group or high-fat diet treated with FV assumption group (Figure 5(b)). Compared with the mouse group fed with HFD, there were 394 DEGs in the mice fed with NFD and 261 in mouse group treated with FV. Next, we also studied the therapeutic effect of FV on NAFLD's main signaling pathways. GO biological process and KEGG enrichment analyses were conducted using WebGestalt.  Figure 5(c) shows the top 10 KEGG pathways which have the most significant false discovery rate (FDR) and *P* value, including PPAR signaling pathway, fatty acid elongation, and biosynthesis of unsaturated fatty acids. According to GO enrichment analysis results, the 10 most significantly enriched GOBP terms are listed in Figure 5(d) (*P* < 0.05). The results suggested that DEGs participated in the regulation of metabolic processes for lipid, small molecule, unsaturated fatty acid, etc.

In addition, the GSEA pathway enrichment results indicated that the cellular signaling pathways related to inflammation (such as the T cell receptor signaling pathways), lipid metabolism (such as the PPAR signaling pathway and biosynthesis of unsaturated fatty acids), and fibrosis (such as the extracellular matrix (ECM) receptor interaction) were enriched and significantly downregulated by FV treatment (Figures 5(e) and 5(f)). The results of RNA-seq showed that lipid metabolism-related genes, such as CD36, had significantly different expressions between the HFD-fed mice treated with FV and the mice without FV treatment. This indicated that the CD36 differential gene may be one of the primary responsive targets that cause the FV to affect HFD-fed mice (Figure 5(g)). Therefore, we hypothesized that potential targets of FV might be present in the CD36 gene of the lipid metabolism signaling pathway. The following experiments were conducted to confirm this hypothesis.

### 3.6. CD36: A Potential Target of FV for Ameliorating Lipid Accumulation

**Figure 6:**
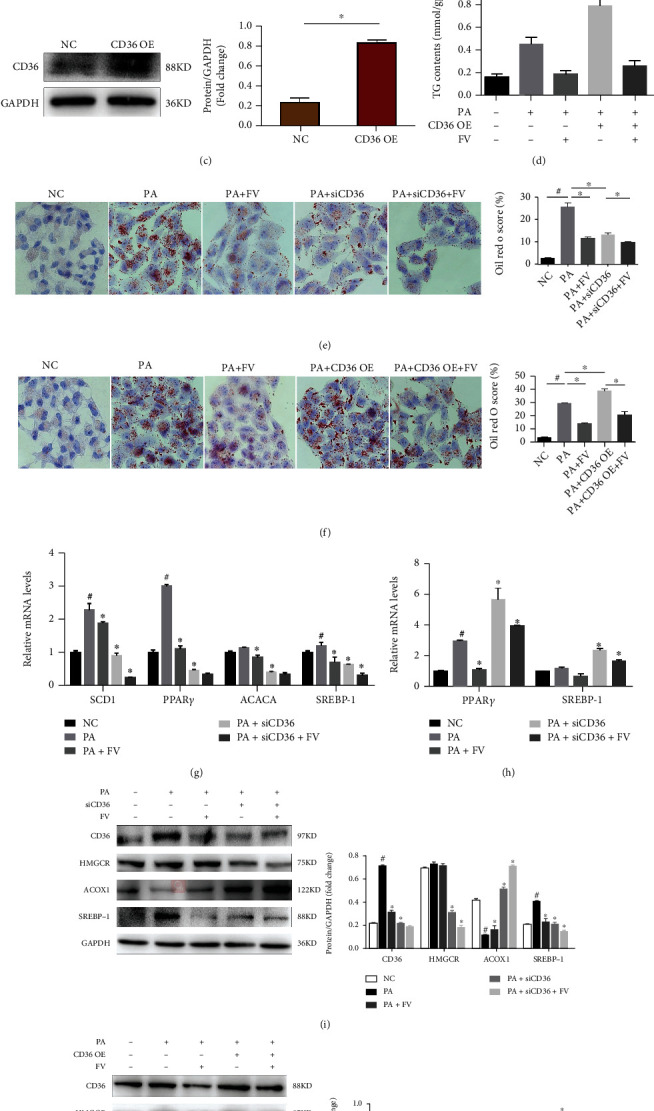
Alleviation of lipid accumulation and inflammation by FV through regulating CD36 in HepG2 cells with PA inducement. (a, c) CD36 protein levels after CD36 knockdown or CD36 overexpression and quantitative analysis. (b, d) TG contents of HepG2 cells in different treatment groups. (e, f) Oil red O to examine the accumulation level of lipid in HepG2 cells and their quantified scores (magnification 100x, scale bar = 250 *μ*m). (g) The mRNA levels of SCD1, PPAR *γ*, ACACA, SREBP-1, IL-1*β*, and CCL5 in siCD36 HepG2 cells with or without FV administration under the condition of PA inducement. (h) The mRNA levels of PPAR *γ*, SREBP-1, IL-1*β*, and TNF-*α* in CD36 OE HepG2 cells with or without FV administration under the condition of PA inducement. (i) The protein levels of CD36, HMGCR, ACOX1, and SREBP-1 in siCD36 HepG2 cells. (j) The protein levels of CD36, HMGCR, ACOX1, and SREBP-1 in CD36 OE HepG2 cells. *n* = 3, ^∗^*P* < 0.05 vs. NC group for (a, c); ^#^*P* < 0.05 vs. NC group, ^∗^*P* < 0.05 vs. PA group or PA+siCD36 group/PA+CD36 OE group (b–j).

We confirmed that FV treatment restored the level of CD36 in the liver of mice, which was significantly elevated by high-fat diet daily. To evaluate the functional role of CD36 in the pathological process of lipid accumulation, we generated plasmid overexpressing CD36 in HepG2 cells (Figure 6(c)) and observed that the overexpression of CD36 significantly increased the lipid accumulation through oil red O staining and TG level; in addition, the overexpression of CD36 was obviously reversed by FV administration (Figures 6(d) and 6(f)). Moreover, after the overexpression of CD36, the levels of PPAR*γ*, SREBP-1, and HMGCR were upregulated and were significantly suppressed by FV treatment (Figures 6(h) and 6(j)). Also, after the overexpression of CD36, the expression of ACOX1 related to fatty acid oxidation was decreased in HepG2 cells and was reversed by FV treatment (Figure 6(j)). We also utilized siRNA to knock down the mRNA level of CD36 in HepG2 hepatocytes (Figure 6(a)). Through oil red O staining and TG level (Figures 6(b) and 6(e)), we observed that siRNAs targeting CD36 significantly attenuated PA-induced lipid accumulation, which was consistent with the role of FV. In addition, lipid metabolism-related proteins, including PPAR*γ*, SCD1, ACACA, SREBP-1, and HMGCR, were downregulated by the FV+siCD36 group (Figures 6(g) and 6(i)), while the expression of ACOX1 was upregulated by siCD36 and FV.

Considering the important role of chronic inflammation in NAFLD, we evaluated the influence of CD36 on the inflammatory response. As shown in Figure 6(h), CD36 overexpression marked an increase in the mRNA expression of IL-1*β* and TNF-*α*, which were ameliorated by FV treatment, while the mRNA expression of IL-1*β* and CCL5 was obviously suppressed by CD36 knockdown (Figure 6(g)).

### 3.7. Modulation of Gut Microbiota Composition in HFD-Fed Mice by FV

**Figure 7:**
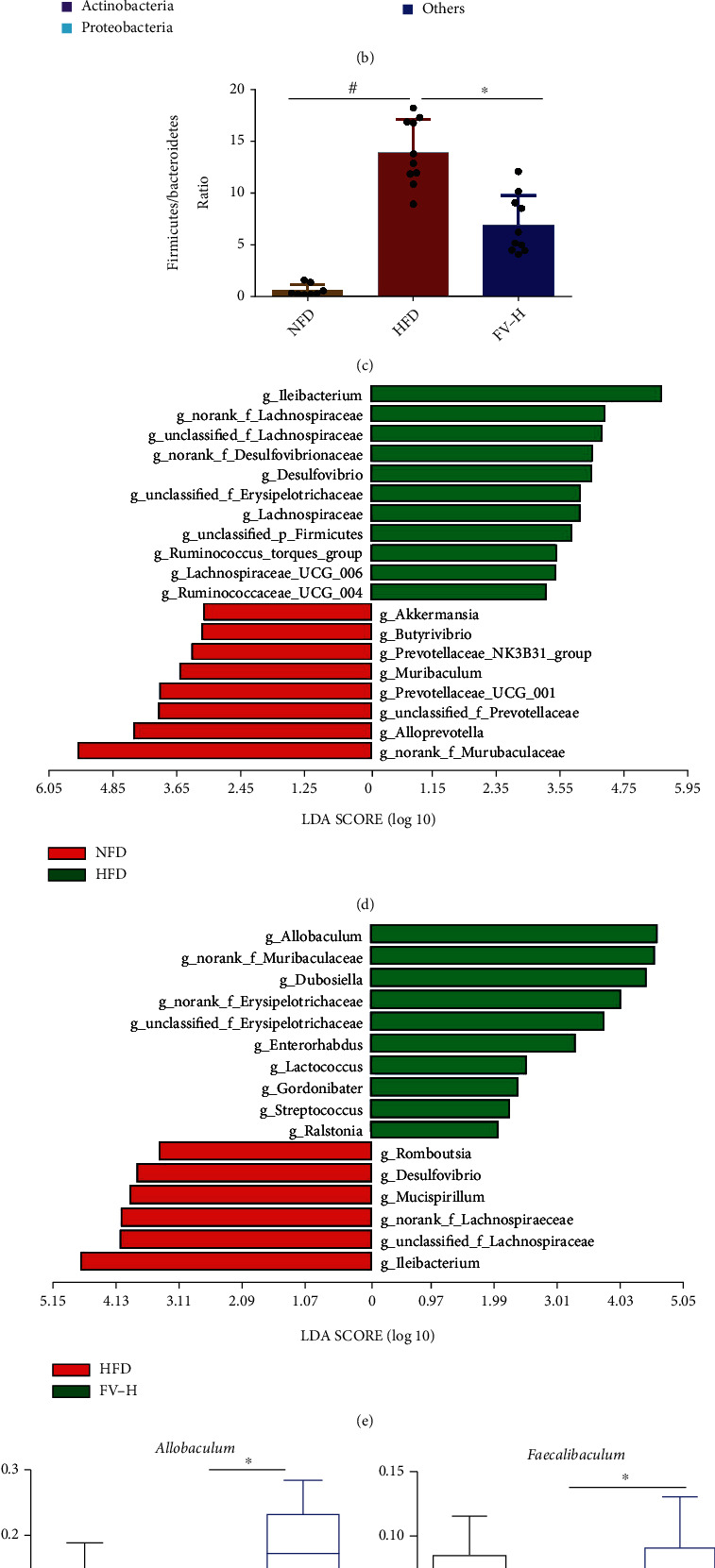
Diversity of gut microbiota in mouse models induced by different diets. (a) PCoA result structure of each group. (b) Taxonomic distribution of bacterial communities of NAFLD mouse fecal samples at the phylum level. (c) *Firmicutes*/*Bacteroidetes* ratio at the phylum level. (d) LDA score of differentially abundant taxa between the NFD group and the HFD group. (e) LDA score of differentially abundant taxa between the HFD group and the FV-H group. (f) Relative abundance of *g_Allobaculum*, *g_Faecalibaculum*, *g_Lactococcus*, *g_norank_f_Muribaculaceae*, *g_Ileibacterium*, *g_Lachnospiraceae_UCG-006*, *g_Desulfovibrio*, and *g_Ruminococcus_torques_group* at the genus levels. *n* = 8, 10, 10; ^#^*P* < 0.05 vs. the NFD group, ^∗^*P* < 0.05 vs. the HFD group.

In order to investigate the effects of FV treatment on the gut microbiota, we used 16S rRNA genetic sequencing to study the composition of the microbiota in mice. From the PCoA results for the gut microbiota, we found that the NFD group, the HFD group, and the FV-H group were clustered into three isolated groups, and the values for the FV-H group were clustered between the other groups (Figure 7(a)). Moreover, the relative abundance of bacteria at the phylum level in the three groups was calculated and shown in Figure 7(b). From the figure, the amount of *Firmicutes* of mice in the HFD group is higher and the amount of *Bacteroidetes* is lower. In addition, the *Firmicutes*/*Bacteroidetes* ratio in the HFD group was higher than that in the NFD group, but lower in the FV-H treatment group (Figure 7(c)). In obese individuals, the *Firmicutes*/*Bacteroidetes* ratio in gut microbiota is usually higher [24]. Therefore, the reduction of *Firmicutes*/*Bacteroidetes* ratio in the FV-H group indicates that FV administration can reverse this parameter of obesity.

Subsequently, in order to determine the changes in specific bacterial taxa after the intervention of FV supplementation, we used the linear discriminant analysis (LDA) effect size (LEfSe) to identify the difference in the fecal microbiota composition between the HFD group and the FV-H group. At the genus level, the LDA score was used to analyze specific taxa in different test groups of the mice (Figures 7(d) and 7(e)). Compared with the NFD group, the abundance of *g_Ileibacterium*, *g_Lachnospiraceae_UCG-006*, g*__Ruminococcus_UCG_004*, and *g_Lachnoclostridium* (Figure 7(d)) in feces of HFD-fed mice was increased, which has been reported to be associated with liver and colon inflammation and relevant mouse or human diseases, including metabolic syndrome, gastrointestinal injury, and immune system disinfection [25, 26]. The same result was obtained in the abundance of *g_Desulfovibrio*, which is a key producer of endotoxins in animal models of obesity (Figure 7(l)) [27]. The FV supplementation significantly reduced these genera (Figure 7(e)). Correspondingly, the abundance of *g_Allobaculum*, *g_Faecalibaculum*, *g_norank_f_Muribaculaceae*, and *g_Butyricicoccus* was increased by FV treatment in HFD-fed mice (Figures 7(f)–7(i)). However, the abundance of *g_Ileibacterium*, *g_Lachnospiraceae_UCG-006*, and *g_Ruminococcus_torques_group* was decreased by FV treatment in HFD-fed mice (Figures 7(j), 7(k), and 7(m)). The bacterium of *g_Allobaculum*, *g_Faecalibaculum*, and *g_Butyricicoccus* is acted as the producer of short-chain fatty acids (SCFAs) in the gut, such as formic acid, acetic acid, propionic acid, butyric acid, and valerate acid. More and more studies have emphasized on SCFAs' alleviating effects on inflammation and protective effects on gut barrier function [28]. It has been proved that supplementing animals with butyrate can reduce liver fat accumulation and liver inflammation [29]. The *g_norank_f_Muribaculaceae* also showed a strong negative correlation with several obesity-related indicators in mice with a high-fat diet [30].

### 3.8. Correlations among the Gut Microbiota, Signaling Pathways, and Metabolic Parameters

**Figure 8:**
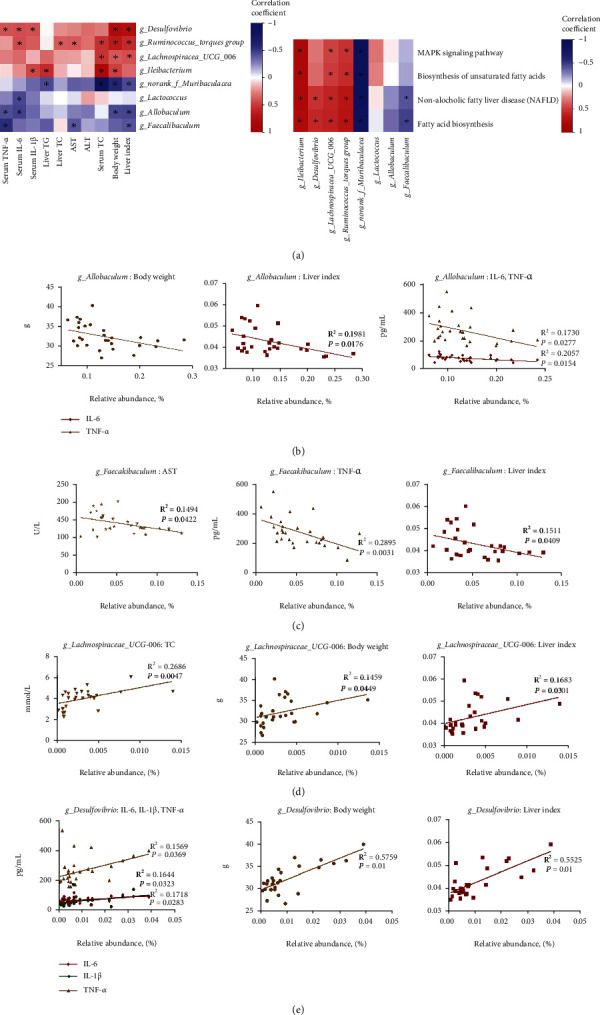
Correlation analyses between metabolic parameters and genera microbiota. (a) Spearman correlation heat map between 8 genera microbiota and 10 metabolic parameters; Spearman correlation heat map between 8 genera microbiota and 4 signaling pathways. (b–e) Linear regression analyses between metabolic parameters and genera microbiota. Significant correlations were marked by ^∗^*P* < 0.05.

In order to study the relationship between the fecal microbiota and the liver pathological condition, we compared 8 bacterial genera and 4 signaling pathways with 10 metabolic parameters using correlation analyses. The results indicated that *Muribaculaceae* had significantly negative correlation with the level of liver index, body weight, serum TC, and liver TG. And *Allobaculum* was negatively correlated with liver index, body weight, serum IL-6, and serum TNF-*α*. In addition, *Faecalibaculum* had an obviously negative correlation with liver index, serum IL-1*β*, and AST, while *Ruminococcus_torques_group* was positively correlated with these parameters. Moreover, *Lachnospiraceae_UCG_006*, *Desulfovibrio*, and *Ileibacterium* were positively correlated with liver index, liver weight, body weight, serum TC, IL-6, IL-1*β*, and TNF-*α* (Figure 8(a)).

Consistently, the results also demonstrated that beneficial bacterium was negatively correlated with the 4 signaling pathways, including NAFLD pathway, MAPK signaling pathway, fatty acid biosynthesis, and biosynthesis of unsaturated fatty acid. In particular, *Muribaculaceae* was significantly negatively correlated with all signaling pathways, and *Faecalibaculum* was positively negatively correlated with NAFLD and fatty acid biosynthesis signaling pathway. However, *Ileibacterium*, *Lachnospiraceae_UCG_006*, and *Ruminococcus_torques_group* were significantly positively correlated with all signaling pathways, and *Desulfovibrio* was significantly positively correlated with NAFLD and fatty acid biosynthesis signaling pathways (Figure 8(a)). As shown in Figures 6(b) and 6(c), *Allobaculum* had a negative linear correlation with IL-6, TNF-a, body weight, and liver index (*P* < 0.05); *Faecalibaculum* had a negative linear correlation with AST, TNF-*α*, and liver index (*P* < 0.05). Moreover, Figures 6(d) and 6(e) show that *Lachnospiraceae_UCG_006* had a positive linear correlation with TC, body weight, and liver index (*P* < 0.05), while *Desulfovibrio* had a positive linear correlation with IL-1*β*, IL-6, TNF-*α*, body weight, and liver index (*P* < 0.05).

## 4. Discussions

The UPLC-Q/TOF-MS/MS identification result of *Ficus hirta* Vahl. showed 54 compounds, including apigenin, luteolin, psoralen, vitexin, and bergamot lactone. Previous studies have found that apigenin can regulate hepatocyte lipid metabolism and oxidative stress by adjusting PPAR*γ* [31], Nrf2 [31], and XO/NLRP3 pathways [32], thereby attenuating HFD-induced NAFLD. Psoralen is one of the main components of FV, which has the ability to relieve lipid accumulation in PA-induced primary hepatocyte model of NAFLD through downregulating the intracellular content of TC and TG [33]. Simultaneously, the 8-methoxypsoralen, a vitamin D receptor ligand with a promising antisteatosis action, can relieve the symptoms of NAFLD by binding to vitamin D receptor [27]. Furthermore, other ingredients, such as naringenin [34], luteolin [35], and bergapten [36], can also improve NAFLD by relieving liver inflammation or regulating lipid metabolism. Therefore, we speculate that FV may play the role of treating NAFLD through the above components.

**Figure 9:**
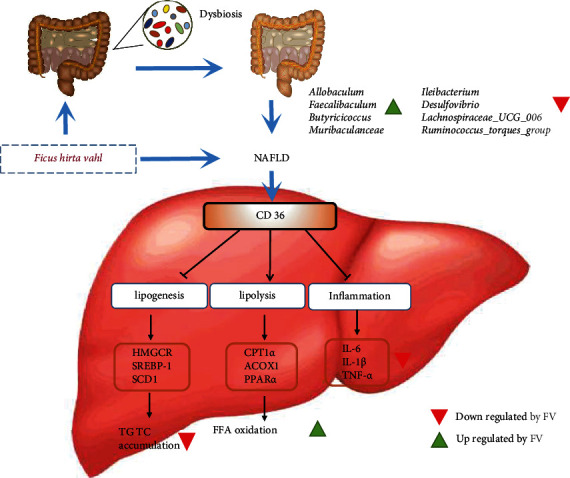
The speculative mechanism of *Ficus hirta* Vahl. for treatment of NAFLD in HFD-fed mice. FV alleviates nonalcoholic fatty liver disease through adjusting lipid metabolism and inflammation via downregulation of CD36 and regulating the structure of gut microbiota.

In this study, our results proved that natural herbs *Ficus hirta* Vahl. can prevent and treat NAFLD using *in vitro* and *in vivo* models. In comparison with the mice in the HFD group, FV administration alleviated obesity, ameliorated the accumulation of lipid, and attenuated liver inflammation and lipogenesis in HFD-fed mice. Moreover, we firstly discovered that CD36 as a potential target of FV for NAFLD and then FV exerted pharmacologic effects against NAFLD partly by reducing the expression of CD36 to improve lipid metabolism and inflammation. Meanwhile, we revealed that the regulation of the gut microbiota structure by FV supplementation can improve liver inflammation in HFD-fed mice. These results have demonstrated that FV may be used as a candidate to treat lipid metabolism including NAFLD and inflammatory disorders in the future (Figure 9).

Accumulation of lipid in hepatocyte is an important indicator for the pathogenesis of NAFLD [37]. In hepatocytes, fatty acids are mainly stored and transported in the form of TG [38]. However, TG can be excessively accumulated in hepatocytes in NAFLD due to the metabolic disorder of fatty acid [39]. Our data have indicated that compared with NFD-fed mice, TG contents of liver tissue increased obviously in HFD-fed mice, as shown in Figure 3(h), which is consistent with the above results. In contrast, FV supplementation alleviated this symptom in a dose-dependent manner, which indicated that FV was a great contributor to suppress the TG accumulation. Besides, the outcome of oil red O staining revealed that FV supplementation remarkably suppressed the formation of lipid droplet in liver tissue of HFD fed mice.

To provide further insights into the role of FV in hepatic lipid metabolism, we studied the expression of genes related to lipid metabolism using both *in vivo* and *in vitro* models. SREBP-1 and its downstream proteins involving ACACA and SCD1 are important transcription factors that regulate the synthesis of fatty acid. CPT1*α*, ACOX1, and PPAR*α* are associated with fatty acid oxidation, and CD36 and FABP1 are related to lipid uptake. Previous study suggested that dioscin can help ameliorate NAFLD by inhibiting the expression of ACACA, SCD1, and SREBP1 of the liver in HFD-fed mice [40], which is in agreement with our results on the effect of FV treatment. Additionally, we found that after FV treatment, the expression of CPT1*α* and PPAR*α* in mice was significantly upregulated. These findings were similar to previous studies [41]. All the outcomes above confirmed that FV contributed to lipid metabolism.

In order to comprehensively reveal the role of FV in NAFLD and the associated mechanism, we conducted RNA-seq analysis of liver tissues in the HFD-fed mice and corresponding diet mice with FV supplementation. GSEA revealed transcriptional levels involved in significant regulation of lipid metabolism, inflammation, and fibrosis. CD36 is a central regulator for cells metabolism, lipid maintenance, and glucose metabolism. In addition, CD36 transduces signals to mediate its role in inflammation [42] and lipid metabolism, thus accelerating the progression of metabolic diseases including obesity, atherosclerosis, NAFLD, and type 2 diabetes [43]. From the RNA-seq results, FV treatment significantly downregulated the mRNA level of CD36, which may use a feed-forward loop to facilitate the entry of fatty acids, thereby providing positive effects on its own de novo synthesis and functioning as a ligand for PPAR*γ*. PPAR*γ* increases the gene expression of essential proteins that support lipid droplet expansion [44] and it is expressed at low levels in normal liver, whereas increased expression of PPAR*γ* is a common feature of hepatic steatosis [45]. Importantly, PPAR*γ* can activate the transcription of CD36. CD36 silencing ameliorates lipid accumulation and improves hepatic steatosis by restoring the reduction in fatty acid oxidation *in vitro* [46]. CD36 has been implicated in inflammatory signaling induced by ox-LDL [47]. CD36 can bind to ox-LDL and activate the JNK signaling pathway to induce inflammation; in addition, CD36 can mediate the production of ROS by activating the NLRP3 inflammasome [48]. Our data proved that CD36 intensified the accumulation of the hepatocyte lipid in NAFLD. Interestingly, FV suppressed the expression and activity of CD36, and FV treatment rescued the exacerbated effects of CD36 on lipid metabolism and inflammation. After the HFD-fed mouse group and PA-induced HepG2 cells were treated with FV, the genes related to lipid synthesis, including SREBP-1, ACACA, and SCD1, were apparently decreased, but the genes involved in fatty acid oxidation, such as CPT1*α*, ACOX1, and PPAR*α*, were significantly elevated. These results indicated that FV could be a potential candidate for NAFLD by attenuating the overaccumulation of lipid.

A few mechanisms have been suggested for the gut microbiome and NAFLD, including the gut microbiome dysbiosis that shifts bacterial components and results in hepatic inflammation. Also, the gut microbiota may produce different metabolites that cause NAFLD susceptibility [49]. In addition, some species of microbes can produce specific enzymes to ferment nutrients into an absorbable form. For example, the conversion of indigestible carbohydrates into SCFAs [50] may have anti-inflammatory and immunomodulatory effects [51]. Otherwise, when bacteria regulate the intestinal permeability, certain species may promote the “leaky gut.” In this case, metabolites related to microbes enter the bloodstream from the gut. Consequently, the body produces cytokines and other mediators to initiate an inflammatory response [52]. Our analysis on the fecal microbiota has shown that FV can improve the gut microbiota dysbiosis in the mice fed with HFD. Also, it is suggested that a high *Firmicutes*/*Bacteroidetes* ratio increases the energy uptake and results in obesity because the members of the phylum *Firmicutes* are more efficient than the members of the *Bacteroidetes* in helping the host obtain calories from food [53]. However, the relative abundance of bacteria at the phylum level revealed that FV significantly reduced *Firmicutes/Bacteroidetes* ratio in comparison with the HFD group.

Many studies have aimed at identifying the specific bacteria changes that lead to NAFLD. In our study, after FV administration, at least eight microbiota genera that reside have been changed in the gut. Among them, *Allobaculum*, *Faecalibaculum*, and *Butyricicoccus* have been identified as SCFA-producing bacterium and are inversely associated with different proinflammatory markers. Butyrate is an anti-inflammatory metabolite with the known inhabitation effect on the producing pathway of proinflammatory cytokines [54]. SCFAs inhibit HDAC (histone deacetylase) activity, promote histone acetylation, affect inflammatory response, and contribute to intestinal homeostasis [55]. As bacterial abundance increased sequentially, *Ileibacterium*, *Lachnospiraceae_UCG_006*, *Desulfovibrio*, and *Ruminococcus_torqus_group* increased with the progression of NAFLD. This phenomenon is in line with the study result on the mouse model that *Desulfovibrio* is strongly correlated with obesity, metabolic syndrome, and inflammation [56]. *Ileibacterium*, a novel member of the family Erysipelotrichaceae, was upregulated by LPS induction [57], but its abundance in HFD-fed mice was downregulated by FV treatment. *Lachnospiraceae_UCG_006* is the main genus of *Lachnospiraceae* and has a positive correlation with the pathological characteristics of colitis [58]. These results have indicated that a high-fat diet may lead to imbalance of the gut microecology and activate intestinal pathogenic bacteria to cope with inflammation, and then, the increase in abundance of beneficial bacteria might be caused by the therapeutic effect of the FV supplementation diet.

In the liver, the mitogen-activated protein kinase (MAPK) signaling pathway is important in regulating metabolism [59], as the obesity and the related inflammatory state in insulin-responsive tissues activate the stress-responsive MAPKs, and the hypothesis that MAPKs signaling pathway drives liver metabolic dysfunction has been accepted [60]. In addition, fatty acids accumulate in the liver by hepatocellular uptake and biosynthesis. The metabolic disorders disrupt the balance of hepatic fatty acid metabolism, thus usually causing the accumulation of TG in the liver and NAFLD [38]. Our research results indicated that FV suppressed the MAPK signaling pathway and fatty acid biosynthesis, thereby attenuating the severity state of NAFLD in HFD-fed mice.

## 5. Conclusion

The results of this study revealed that FV treatment can commendably ameliorate lipid metabolism and hepatic inflammation by regulating CD36 and alleviate the progression of NAFLD by regulating the composition and potential function of the gut microbiota. *Ficus hirta* Vahl. is an ideal medicine to improve the pathophysiology of diet-induced metabolic diseases and NAFLD.

## Figures and Tables

**Table 1:** Compounds identified in FV prescription by UPLC-Q/TOF-MS.

No.	Retention time (min)	Molecular formula	[M+H]^+/-^(m/z) (mass error) (ppm)	Intensity	Identification
1	0.6	C_6_H_9_N_3_O_2_	156.077(1.7) [M+H]^+^	131400	Histidine
2	0.65	C_5_H_9_NO_2_	116.0707(0.7) [M+H]^+^	731900	Proline
3	0.67	C_7_H_7_NO_2_	138.0553(2.6) [M+H]^+^	692800	Trigonelline
4	0.68	C_7_H_12_O_6_	191.0559(-0.9) [M+H]^−^	1043000	Quinic acid
5	0.77	C_4_H_6_O_5_	133.0139(-2.5) [M+H]^−^	1208000	L-Malic acid
6	0.94	C_6_H_8_O_7_	191.019(-3.6) [M+H]^−^	3592000	Citric acid
7	0.95	C_5_H_5_N_5_	136.0621(2.4) [M+H]^+^	500200	Adenine
8	1.05	C_6_H_5_NO_2_	124.0391(-1.9) [M+H]^+^	46600	Nicotinic acid
9	1.1	C_8_H_11_NO_3_	170.0813(0.5) [M+H]^+^	53320	Vitamin B6
10	1.2	C_9_H_12_N_2_O_6_	243.062(-1.1) [M+H]^−^	1073000	Uridine
11	1.28	C_4_H_6_O_4_	117.0203(8.6) [M+H]^−^	162400	Amber acid
12	1.4	C_6_H_13_NO_2_	132.102(0.4) [M+H]^+^	24570	Leucine
13	1.65	C_10_H_15_NO	166.1225(-0.7) [M+H]^+^	4021000	Hordenine
14	2.06	C_10_H_13_N_5_O_5_	284.0993(1.3) [M+H]^+^	115600	Guanosine
15	2.27	C_10_H_13_N_5_O_4_	266.0892(-0.9) [M+H]^−^	172100	Adenosine
16	2.52	C_9_H_11_NO_2_	164.0716(-0.4) [M+H]^−^	144900	Phenprobamate
17	2.78	C_6_H_6_O_3_	127.0393(2.3) [M+H]^+^	170000	5-Hydroxymethylfurfural
18	3.93	C_17_H_26_O_11_	407.1546(-0.6) [M+H]^+^	408900	8-O-Acetylharpagide
19	5.15	C_21_H_18_O_12_	461.0723(-0.5) [M+H]^−^	14410	Luteolin-7-O-*β*-D-glucuronide
20	5.24	C_7_H_6_O_3_	137.0244(-0.2) [M+H]^−^	602000	Protocatechuic aldehyde
21	7.38	C_15_H_14_O_6_	289.0716(-0.6) [M+H]^−^	351700	Catechin
22	7.5	C_7_H_6_O_2_	121.0305(8.1) [M+H]^−^	495700	p-Hydroxybenzaldehyde
23	8.19	C_8_H_8_O_2_	137.0602(3.8) [M+H]^+^	347200	Anisaldehyde
24	8.46	C_8_H_8_O_4_	167.035(0) [M+H]^−^	250800	Vanillic acid
25	8.53	C_9_H_6_O_4_	177.0195(0.7) [M+H]^−^	134600	Esculetin
26	10.31	C_8_H_8_O_2_	135.0448(-2.5) [M+H]^−^	321000	4′-Hydroxyacetophenone
27	12.48	C_9_H_6_O_3_	161.0238(-3.7) [M+H]^−^	794100	4-Hydroxycoumarin
28	12.51	C_9_H_8_O_3_	163.04(-0.3) [M+H]^−^	163500	p-Coumaric acid
29	12.67	C_9_H_10_O_4_	183.0653(0.5) [M+H]^+^	160400	Syringaldehyde
30	12.8	C_21_H_20_O_12_	463.0875(-0.6) [M+H]^−^	27330	Quercetin-3′-O-glucoside
31	12.9	C_9_H_6_O_3_	163.039(0.2) [M+H]^+^	204600	7-Hydroxycoumarin
32	12.96	C_10_H_10_O_4_	193.0504(-0.9) [M+H]^−^	284200	Ferulic acid
33	13.07	C_10_H_10_O_4_	195.0659(3.4) [M+H]^+^	113500	Isoferulic acid
34	13.89	C_10_H_8_O_3_	175.04(-0.3) [M+H]^−^	192700	7-Methoxycoumarin
35	14.63	C_15_H_12_O_7_	303.0509(-0.4) [M+H]^−^	248600	Dihydroquercetin
36	16.14	C_27_H_30_O_15_	595.1647(-1.7) [M+H]^+^	48030	Glucosylvitexin
37	18.02	C_21_H_20_O_10_	431.098(-0.8) [M+H]^−^	1645000	Vitexin
38	18.34	C_15_H_12_O_6_	287.0562(0.4) [M+H]^−^	335100	Aromadendrin
39	19.51	C_26_H_28_O_14_	563.1411(0.9) [M+H]^−^	237000	Schaftoside
40	21.9	C_20_H_17_NO_4_	336.123(0) [M+H]^+^	39780	Berberine
41	22.12	C_11_H_6_O_4_	201.0194(0.4) [M+H]^−^	726600	Xanthotoxol
42	22.25	C_11_H_6_O_3_	187.0388(-0.9) [M+H]^+^	36910000	Psoralen
43	24.45	C_15_H_12_O_5_	271.0611(-0.4) [M+H]^−^	777300	Naringenin
44	25.31	C_12_H_8_O_4_	217.0496(0.1) [M+H]^+^	20270000	Bergapten
45	25.32	C_15_H_10_O_6_	285.0402(-0.9) [M+H]^−^	2446000	Luteolin
46	26.65	C_15_H_10_O_5_	269.0455(-0.3) [M+H]^−^	6317000	Apigenin
47	27.92	C_15_H_22_O_2_	235.1694(0.7) [M+H]^+^	1094000	Curcumenol
48	29.08	C_14_H_14_O_3_	229.087(-0.2) [M+H]^−^	476900	Demethylsuberosin
49	29.25	C_15_H_24_O_2_	237.1848(-0.5) [M+H]^+^	2528000	Curdione
50	29.59	C_16_H_14_O_4_	269.082(0.2) [M+H]^−^	654000	Isoimperatorin
51	30.3	C_15_H_20_O_2_	233.1541(2.3) [M+H]^+^	145700	Isoalantolactone
52	35.37	C_16_H_30_O_2_	253.2174(0.4) [M+H]^−^	175800	Sclareol glycol
53	35.57	C_33_H_40_N_2_O_9_	609.2803(-0.6) [M+H]^+^	113300	Reserpine
54	35.75	C_18_H_32_O_2_	279.2332(1) [M+H]^−^	337800	Linoleic acid

## Data Availability

Data can be obtained from the corresponding author. Please email at mailto:s19861020@jnu.edu.cn.

## Abbreviations

NAFLD: Nonalcoholic fatty liver disease
CD36: Cluster of differentiation 36
PA: Palmitic acid
HFD: High-fat diet
TC: Total cholesterol
TG: Total triglyceride
ALT: Alanine aminotransferase
AST: Aspartate aminotransferase
LDL-C: Low-density lipoprotein cholesterol
H&E: Hematoxylin and eosin
ELISA: Enzyme-linked immunosorbent assay
IL-6: Interleukin-6
IL-1*β*: Interleukin-1*β*
TNF-*α*: Tumor necrosis factor-*α*
SREBP-1: Sterol regulatory element-binding protein 1
CPT1*α*: Recombinant carnitine palmitoyltransferase 1*α*
HMGCR: Recombinant 3-hydroxy-3-methylglutaryl coenzyme A reductase
ACOX1: Acyl-coenzyme A oxidase 1
ACACA: Acetyl-coenzyme A carboxylase alpha
FABP1: Fatty acid-binding protein 1
PPAR*α*: Peroxisome proliferator-activated receptor *α*
PPAR*γ*: Peroxisome proliferator-activated receptor *γ*
DEGs: Differentially expressed genes.

## Supplementary Materials

Supplementary Materials Supplementary Table 1 shows the time program of the gradient elution in UPLC. Supplementary Table 2 lists the parameters in the mass spectrometric analysis method. Supplementary Table 3 displays human and mouse primer sequences for quantitative real-time PCR.
