# Supplementary material for: Ficus hirta Vahl. Ameliorates Nonalcoholic Fatty Liver Disease through Regulating Lipid Metabolism and Gut Microbiota
Source: Oxid Med Cell Longev. 2022 May 10;2022:3474723. doi: 10.1155/2022/3474723 (PMC9113867; doi:10.1155/2022/3474723)
Supplement: Supplementary Materials — Supplementary Table 1 shows the time program of the gradient elution in UPLC. Supplementary Table 2 lists the parameters in the mass spectrometric analysis method. Supplementary Table 3 displays human and mouse primer sequences for quantitative real-time PCR. [file 3474723.f1.docx]

Supplementary Table 1 The time program of the gradient elution

| *Time [min]* | *Flow [mL/min]* | *A.Conc [%]* | *B.Conc [%]* | *B.Curve* |
| --- | --- | --- | --- | --- |
| 0.00 | 0.4000 | 90.0 | 10.0 | 0 |
| 0.50 | 0.4000 | 90.0 | 10.0 | 0 |
| 20.00 | 0.4000 | 60.0 | 40.0 | 0 |
| 35.00 | 0.4000 | 5.0 | 95.0 | 0 |
| 40.00 | 0.4000 | 5.0 | 95.0 | 0 |
| 40.10 | 0.4000 | 90.0 | 10.0 | 0 |
| 45.00 | 0.4000 | 90.0 | 10.0 | 0 |

Supplementary Table 2 MS Method Parameters

| **General** |  |
| --- | --- |
| *Method duration (minutes):* | 45 |
| *Total scan time (seconds):* | 0.767 |
| *Estimated cycles:* | 3522 |
| *Actual method duration (minutes):* | 45.48 |
| *Intact protein mode:* | False |
| *Decrease detector voltage:* | False |
| *Large protein (>70kDa):* | False |
| **Ion Source** |  |
| *Source name:* | TurboIonSpray |
| *Curtain gas (psi):* | 35 |
| *Ion source gas 1 (psi):* | 55 |
| *Ion source gas 2 (psi):* | 55 |
| *Temperature (°C):* | 500 |
| **Experiment** |  |
| *IDA Survey:* |  |
| *Scan type:* | TOFMS |
| *Polarity:* | Positive/Negative |
| *Ionspray voltage (V):* | 5500 |
| *CAD gas:* | 8 |
| *TOF start mass (Da):* | 100 |
| *TOF stop mass (Da):* | 1800 |
| *Accumulation time (s):* | 0.1 |
| *Declustering potential (V):* | 80 |
| *Declustering potential spread (V):* | 0 |
| *Collision energy (V):* | 10 |
| *Collision energy spread (V):* | 0 |
| *Time bins to sum:* | 4 |
| *Channel 1:* | True |
| *Channel 2:* | True |
| *Channel 3:* | True |
| *Channel 4:* | True |

Supplementary Table 3 Human and mouse primer sequences for quantitative real-time PCR

| **Gene** | **Forward (5'-3' sequence)** | **Reverse (3'-5' sequence)** |
| --- | --- | --- |
| **Human** |  |  |
| **SCD1** | TCATAATTCCCGACGTGGCT | CCCAGAAATACCAGGGCACA |
| **CD36** | AAAATGTAACCCAGGACG | CACAGCCAGATTGAGAAC |
| **FABP1** | GTCAGTCGTGAAGAGGGAGC | TGCTTCCCATTCTGCACGAT |
| **HMGCR** | ATAGGAACGGTGGGTGGT | CCAATGCTGCCATAAGTG |
| **ACACA** | TCACACCTGAAGACCTTAAAGCC | AGCCCACACTGCTTGTACTG |
| **CCL5** | TCATTGCTACTGCCCTCTGC | TACTCCTTGATGTGGGCACG |
| **IL-1β** | TCGCCAGTGAAATGATGGCT | TGGAAGGAGCACTTCATCTGTT |
| **TNFα** | CGAGTCTGGGCAGGTCTA | AGGGTGTCTGAAGGAGGG |
| **PPARγ** | TACTGTCGGTTTCAGAAATGCC | GTCAGCGGACTCTGGATTCAG |
| **SREBP-1** | TTGCCGACCCTGGTGAGT | ATGGCGTTGTGGGCTGTGC |
| **GAPDH** | ACCCAGAAGACTGTGGATGG | CACATTGGGGGTAGGAACAC |
| **Gene** | **Forward (5'-3' sequence)** | **Reverse (3'-5' sequence)** |
| **Mouse** |  |  |
| **Acaca** | GGCCAGTGCTATGCTGAGAT | AGGGTCAAGTGCTGCTCCA |
| **Cd36** | GACTGGGACCATTGGTGATGA | AAGGCCATCTCTACCATGCC |
| **Cpt1α** | AGGACCCTGAGGCATCTATT | ATGACCTCCTGGCATTCTCC |
| **Srebp-1** | GGAGAACCTGACCCTACGA | CACCACTTCGGGTTTCAT |
| **Hmgcr** | GGACCAACCTTCTACCTC | GCTCACCAGCCATCACAG |
| **Pparα** | CTGTGGGCTCACTGTTCT | AGGGCTCATCCTGTCTTT |
| **Pparγ** | ATTCTGGCCCACCAACTTCGG | TGGAAGCCTGATGCTTTATCCCCA |
| **Scd1** | GTTCCGCCACTCGCCTACA | CACCGTCTTCACCTTCTC |
| **IL-β** | AATGCCACCTTTTGACAGTGATG | AGCTTCTCCACAGCCACAAT |
| **Tnfα** | AGGGTCTGGGCCATAGAACT | CCACCACGCTCTTCTGTCTAC |
| **Ccl5** | ACCACTCCCTGCTGCTTT | ACACTTGGCGGTTCCTTC |
| **Gapdh** | TGTTTCCTCGTCCCGTAG | CAATCTCCACTTTGCCACT |
